# What do Cochrane systematic reviews say about ultrasound-guided vascular access?

**DOI:** 10.1590/1516-3180.2019.0113070519

**Published:** 2019-08-29

**Authors:** Gabriela Araújo Attie, Carolina Dutra Queiroz Flumignan, Melissa Andreia de Moraes Silva, Edivando de Moura Barros, Raul Muffato Daolio, Henrique Jorge Guedes, José Carlos Costa Baptista-Silva, Jorge Eduardo de Amorim, Luis Carlos Uta Nakano, Ronald Luiz Gomes Flumignan

**Affiliations:** I Undergraduate Student Researcher, Department of Surgery, Division of Vascular and Endovascular Surgery, Escola Paulista de Medicina (EPM), Universidade Federal de São Paulo (UNIFESP), São Paulo (SP), Brazil.; II MD, PhD. Researcher, Department of Surgery, Division of Vascular and Endovascular Surgery, Escola Paulista de Medicina (EPM), Universidade Federal de São Paulo (UNIFESP), São Paulo (SP), Brazil.; III MD. Researcher, Postgraduate Program on Interdisciplinary Surgical Science, Escola Paulista de Medicina (EPM), Universidade Federal de São Paulo (UNIFESP), São Paulo (SP), and Division of Vascular and Endovascular Surgery, Hospital de Clínicas de Itajubá, Itajubá, MG, Brazil.; IV Undergraduate Student Researcher, Department of Surgery, Division of Vascular and Endovascular Surgery, Escola Paulista de Medicina (EPM), Universidade Federal de São Paulo (UNIFESP), São Paulo (SP), Brazil.; V Undergraduate Student Researcher, Department of Surgery, Division of Vascular and Endovascular Surgery, Escola Paulista de Medicina (EPM), Universidade Federal de São Paulo (UNIFESP), São Paulo (SP), Brazil.; VI MD, PhD. Adjunct Professor, Department of Surgery, Division of Vascular and Endovascular Surgery, Escola Paulista de Medicina (EPM), Universidade Federal de São Paulo (UNIFESP), São Paulo (SP), Brazil.; VII MD, PhD. Full Professor and Chief, Department of Surgery, Division of Vascular and Endovascular Surgery, Escola Paulista de Medicina (EPM), Universidade Federal de São Paulo (UNIFESP), São Paulo (SP), Brazil.; VIII MD, PhD. Adjunct Professor, Department of Surgery, Division of Vascular and Endovascular Surgery, Escola Paulista de Medicina (EPM), Universidade Federal de São Paulo (UNIFESP), São Paulo (SP), Brazil.; IX MD, PhD. Adjunct Professor, Department of Surgery, Division of Vascular and Endovascular Surgery, Escola Paulista de Medicina (EPM), Universidade Federal de São Paulo (UNIFESP), São Paulo (SP), Brazil.; X MD, PhD. Adjunct Professor, Department of Surgery, Division of Vascular and Endovascular Surgery, Escola Paulista de Medicina (EPM), Universidade Federal de São Paulo (UNIFESP), São Paulo (SP), Brazil.

**Keywords:** Ultrasonography, Punctures, Review, Evidence-based medicine, Vascular access devices

## Abstract

**BACKGROUND:**

Ultrasonography is currently used in investigating many vascular diseases, especially for guiding vascular access.

**OBJECTIVE:**

The objective here was to summarize the evidence from Cochrane systematic reviews (SRs) on the effects of ultrasound-guided vascular access as an intervention approach.

**DESIGN AND SETTING:**

Review of SRs, conducted in the Division of Vascular and Endovascular Surgery of Universidade Federal de São Paulo.

**METHODS:**

A broad search was conducted in the Cochrane Database of Systematic Reviews to retrieve any Cochrane SRs that assessed the effects of ultrasound guidance as a therapeutic approach towards performing any vascular access. The key characteristics and results of all the reviews included were summarized and discussed.

**RESULTS:**

Three SRs on venous access at all ages and one review on arterial access in pediatric participants were included. There was low to moderate certainty of evidence that ultrasound increased the success rate from the first puncture and the overall success rate of the procedure; and reduced the total rate of perioperative and postoperative adverse events, number of punctures, time needed to achieve success and rate of failure to place catheters.

**CONCLUSION:**

Evidence of low to moderate quality showed that ultrasound-guided vascular access seems to reduce the total rate of perioperative and postoperative complications/adverse effects, number of punctures, time needed to achieve success and rate of failure to perform venous catheterization in adults and arterial punctures in children. There is a lack of information regarding ultrasound-guided arterial puncture in adults. Further studies are still imperative for reaching solid conclusions, especially regarding arterial ultrasound-guided access.

## INTRODUCTION

In almost all medical specialties, from pediatrics to geriatrics, at some point, doctors face the need to use vascular access in their patients. In the United States, over 15 million central vascular catheter-days occur in intensive care units per year.^1^ Anatomical landmarks have been used as a guide for performing vascular access for a long time, but their use has been correlated with a number of complications (e.g. infections, hematomas, pneumothorax and death).^1^^,^^2^^,^^3^

Over recent decades, ultrasound has been used as a possible aid for diagnostic purposes, including in bedside examinations and for possibly avoiding complications in various procedures.^4^ In addition, technological advances have made portable ultrasound viable.^4^^,^^5^^,^^6^

Although there are some practical guidelines that make recommendations regarding standard use of ultrasound to guide venous catheterization, up to 40% of doctors are resistant to using this evidence in their practice.^7^^,^^8^ A number of guidelines are available for evaluating ultrasound-guide venous access but there is a lack of such guidelines for arterial sites.^9^^,^^10^

Despite the large amounts of money that have been invested in research on this topic, the relevance of ultrasound-guided vascular access as a therapeutic approach is still a matter of debate, especially in relation to arterial puncture. Because the use of ultrasound as an additional intervention may be a reasonable alternative for improving the results relating to many types of vascular access, it is imperative to assess the effects of ultrasound-guided access through well-conducted randomized controlled trials.

## OBJECTIVE

The aim of this study was to identify and summarize the evidence from Cochrane systematic reviews (SRs) regarding ultrasound-guided vascular access, in an overview.

## METHODS

### Design and setting

This was a review of Cochrane systematic reviews conducted in the Division of Vascular and Endovascular Surgery, Universidade Federal de São Paulo, Brazil.

### Inclusion criteria

#### Types of study

Full Cochrane SRs published in the Cochrane Database of Systematic Reviews (CDSR) were included, without restrictions regarding date of publication. Withdrawn or outdated versions of SRs and protocols for SRs were considered not relevant.

#### Types of participants

We considered all participants who underwent a vascular access procedure, both males and females, of all ages, without any restriction regarding the site of puncture.

#### Types of interventions

We considered SRs that assessed any vascular access technique, such as the Seldinger technique, as an intervention, if comparison with ultrasound-guided access was made in at least one of the study arms.^11^

#### Types of outcomes

We considered any patient-relevant clinical or laboratory outcomes, as assessed by the authors of the SRs included.

#### Search for reviews

We conducted a sensitive systematic search in the Cochrane Database of Systematic Reviews (CDSR, via Wiley) on July 3, 2018. We used the following MeSH terms and all related variants in the titles, abstracts and keywords: “vascular access devices”, “endovascular procedures”, “ultrasonography”, “ultrasonography, Doppler” and “ultrasonography, interventional”. The detailed search strategy is presented in Table 1.

**Table 1.** Search strategy and results from the Cochrane Database of Systematic Reviews

Lines	Search terms	Number of records
#1	MeSH descriptor: [Vascular Access Devices] explode all trees	241
#2	MeSH descriptor: [Endovascular Procedures] explode all trees	8,508
#3	MeSH descriptor: [Ultrasonography] explode all trees	13,655
#4	MeSH descriptor: [Ultrasonography, Doppler] explode all trees	2,959
#5	MeSH descriptor: [Ultrasonography, Interventional] explode all trees	1,658
#6	(Device Vascular Access) or (Port Catheter*) or (Venous Reservoir*) or (Vascular Access Port*) or (Vascular Catheter*) or (Intra Arterial Line*) or (Intra-Arterial Line*) or (Arterial Line*) or (Port-A-Cath) or (Port A Cath) or (PortACath) or (Endovascular Procedure*) or (Intravascular Procedure*) or (Intravascular Technique*) or (Endovascular Technique*)	5,048
#7	(Echography) or (Ultrasound Imaging*) or (Ultrasonic Imaging) or (Sonography Medical) or (Diagnostic Ultrasound*) or (Echotomography) or (Diagnos* Ultrasonic) or (Echotomography Computer) or (Tomography Ultrasonic) or (Doppler Ultrasound*) or (Doppler Ultrasonography) or (Doppler Ultrasound Imaging*) or (Ultrasound Interventional) or (Interventional Ultrasonography) or (Ultrasonography Intravascular)	9,818
#8	#1 or #2 or #6	12,812
#9	#3 or #4 or #5 or #7	20,258
#10	#8 and #9	1,425
#11	Filter: in Cochrane Reviews	221

#### Selection of reviews

Two researchers (GAA and RLGF) independently evaluated the titles and abstracts to analyze whether the SRs fulfilled the inclusion criteria. Any disagreement was resolved by consulting other authors (CDQF, MAMS, EMB, HJGN, LCUN, JCCBS and JEA). The SRs were selected and summarized by two authors (GAA and RLGF).

#### Presentation of results

The results from the search and the SRs included were presented as a qualitative synthesis (descriptive approach).

## RESULTS

### Search results

Our search strategy retrieved 221 references and, after screening the titles and abstracts, 11 SRs were preselected. After assessing the full texts, four reviews fulfilled the inclusion criteria and were included in the qualitative synthesis.^12^^,^^13^^,^^14^^,^^15^

### Reviews included

The latest versions of all the SRs included were published between 2011 and 2016. Details regarding the characteristics of the interventions, comparisons and outcomes and the certainty of evidence are presented in Table 2.

**Table 2.** Characteristics of interventions, comparisons, participants and main findings and quality of evidence, as evaluated by means of grading of recommendations, assessment, development and evaluation (GRADE)

Interventions	Comparisons	Participants	Main findings	GRADE
Real-time 2D ultrasound guidance^12^	Anatomical landmarks	Adults and children requiring venous hemodialysis catheter Central venous catheter for non-dialysis indications and studies using audio Doppler ultrasound techniques were excluded	Favored ultrasound guidance: ● reduction of 89% (overall) and 60% (first attempt) in the risk of catheter placement failure (7 studies, 830 catheters) ● reduction of 87% in the risk of arterial puncture (6 studies, 535 catheters) and of 78% in the risk of hematomas (4 studies, 323 catheters) ● reduction of time taken to achieve successful vein puncture (mean 1.4 minutes less)	N.A.
Real-time 2D and Doppler ultrasound guidance^13^	Anatomical landmarks	Adults and children requiring insertion of a central venous catheter via the internal jugular veins	Favored 2D ultrasound guidance: ● reduction of 71% in the rate of total complications overall (14 trials, 2,406 participants) ● reduction of the number of participants with an inadvertent arterial puncture by 72% (22 trials, 4,388 participants) ● overall success rates increased by 12% (23 trials, 4,340 participants) ● mean number of attempts until achieving success in the intervention groups was 1.19 lower (16 trials, 3,302 participants) ● increase in the chance of success at the first attempt by 57% (18 trials, 2,681 participants) ● reduction of the chance of hematoma formation by 73% (13 trials, 3,233 participants) ● decrease in the time taken for successful cannulation by 30.52 seconds (20 trials, 3,451 participants) Results for Doppler ultrasound techniques versus anatomical landmark were uncertain: ● increase in the chance of success at the first attempt by 58% (four trials, 199 participants) ● evidence of no difference regarding all other outcomes	● very low ● low ● very low ● very low ● moderate ● very low ● very low ● low ● very low to moderate
Real-time 2D and Doppler ultrasound guidance^14^	Anatomical landmarks	Adults and children requiring insertion of a central venous catheter via the subclavian veins	Favored 2D ultrasound guidance: ● decrease in arterial puncture by 79% (3 studies, 498 participants) ● reduction of the chance of complications by 71% (6 trials, 1,058 participants) Evidence of no difference regarding 2D and Doppler ultrasound guidance together: ● total number of complications overall ranged from 77% lower to 17% higher (RR 0.52; 6 trials, 1,478 participants) ● overall success rate ranged from 3% lower to 13% higher (RR 1.05; 8 trials, 1,809 participants) Evidence of no difference regarding 2D ultrasound guidance: ● number of attempts until achieving success in the intervention groups ranged from 1.26 lower to 0.5 higher (mean 0.38 lower; 2 trials, 471 participants) ● time taken to achieve successful cannulation in the intervention groups ranged from 56.92 seconds lower to 77.87 seconds higher (mean 10.48 seconds higher; 2 trials, 471 participants) ● success at first attempt ranged from reduction by 15% to increase by 36% (RR 1.08; 2 studies, 115 participants)	● low ● very low ● very low ● low ● very low ● low ● high
Real-time 2D and Doppler ultrasound guidance^14^	Anatomical landmarks	Adults and children requiring insertion of a central venous catheter via the femoral veins	Favored 2D ultrasound guidance: ● increase in overall success rates ranged from none to 23% higher (RR 1.11, 4 studies, 311 participants) ● increased success at first attempt in 73% (3 studies, 224 participants) Evidence of no difference regarding 2D ultrasound guidance: ● risk of arterial puncture ranged from 86% lower to 16% higher (RR 0.4; 4 studies, 311 participants) ● complications rate ranged from 89% lower to 212% higher (RR 0.49; 4 trials, 311 participants) No data available regarding Doppler ultrasound guidance	● moderate ● high ● low ● low N.A.
Real-time 2D ultrasound guidance^15^	Other techniques (palpation/ Doppler ultrasound)	Children (one month to 18 years of age) undergoing arterial line placement	Favored 2D ultrasound guidance: ● increase in first-attempt catheter placement success in 96% (4 studies, 404 participants) ● reduction of the chance of complications (hematoma or ischemia) by 80% (2 studies, 222 participants) ● increase in successful cannulation within the first two attempts in 78% (2 studies, 134 participants)	● moderate ● moderate ● moderate

N.A. = not available; 2D = two-dimensional; RR = relative risk.

### 1. Ultrasound use for placement of hemodialysis catheters

The objective of this systematic review^12^ was to compare real-time two-dimensional (2D) ultrasound venous imaging and the traditional “blind” landmark method for guidance of percutaneous central venous dialysis catheter insertion. Seven studies were identified, enrolling 767 patients and 830 catheter insertions. In almost all the studies, there was no significant heterogeneity. The review authors did not use grading of recommendations, assessment, development and evaluation (GRADE) for classifying the certainty of evidence.

#### Main findings

Real-time 2D ultrasound guidance significantly decreased the overall risk of catheter placement failure (risk relative, RR: 0.11; 95% confidence interval, CI: 0.03 to 0.35) and the risk of catheter placement failure on the first attempt (RR 0.40; 95% CI: 0.30 to 0.52). Use of ultrasound guidance correlated with notably fewer attempts/catheter placements (mean difference (MD) -0.35; 95% CI -0.54 to -0.16).

A meaningful reduction in the time required for successful vein puncture, from the time when the skin was anesthetized, was found with real-time ultrasound guidance (MD -1.40 minutes; 95% CI: -2.17 to -0.63).

#### Complications

Real-time ultrasound guidance was found to significantly decrease the risk of carotid artery puncture (RR 0.22; 95% CI: 0.06 to 0.81) and led to a significant reduction in the risk of hematoma (RR 0.27; 95% CI: 0.08 to 0.88). There were no differences between patient groups regarding the risk of pneumothorax or hemothorax (RR 0.23; 95% CI: 0.04 to 1.38).

#### Conclusions of this study

There are benefits from the use of real-time 2D Doppler ultrasound guidance with regard to the number of catheters successfully inserted on the first attempt. There was lower risk of arterial puncture and hematomas and less time was taken for successful vein puncture.

### 2. Ultrasound guidance versus anatomical landmarks for internal jugular vein catheterization

This systematic review^13^ had the primary objective of evaluating the safety and effectiveness of guided puncture by means of 2D imaging ultrasound or Doppler ultrasound, for insertion of central venous catheters via the internal jugular vein. As secondary objectives, the review authors assessed differences in disclosure using 2D ultrasound or Doppler ultrasound; the effects of ultrasound use during the puncture (real-time or direct) versus the use of ultrasound only for the identification and marking of the vein before the procedure (indirect); and whether the effects were different between distinct groups of patients or between different levels of experience among the professionals who inserted the catheter. Thirty-five studies were included, totaling 5,108 participants.

According to the review authors, almost all of the studies selected for the review had high risk of bias, and the meta-analysis had substantial heterogeneity. The results were presented as comparisons of landmark versus 2D ultrasound, and landmark versus Doppler ultrasound.

#### Main findings

Use of 2D ultrasound improved the overall success rate by 12% (23 trials with 4,340 participants; RR 1.12; 95% CI: 1.08 to 1.17; P-value < 0.00001; I² = 85%), with no difference between use of Doppler and use of 2D ultrasound. However, the quality of this evidence was very low, due to uncertainty regarding the analysis on data from Doppler ultrasound.

The 2D ultrasound reduced the number of attempts needed to succeed (16 studies; 3,302 participants), with very low quality of evidence. Only at the first attempt was it found that Doppler ultrasound had better results (2 studies; 69 participants; RR 1.58; 95% CI: 1.02 to 2.43; P-value 0.04; I² = 57%), with low quality of evidence.

The time taken for successful cannulation was lower with use of 2D ultrasound (20 studies; 3,451 participants; MD -30.52 seconds; 95% CI: -55.21 to -5.82; P-value 0.02; I² = 97%), with very low quality of evidence. There was no evidence of difference in this outcome using Doppler ultrasound (5 studies; 214 participants; MD 62.04 seconds; 95% CI: -13.47 to 137.55; P-value 0.11; I² = 50%), with moderate quality of evidence.

#### Complications

Use of 2D ultrasound reduced the total number of perioperative and postoperative complications by 71% (14 trials with 2,406 participants; RR 0.29; 95% CI; 0.17 to 0.52; P-value < 0.0001; I² = 57%). However, the quality of evidence was very low. There was no difference when Doppler ultrasound was used instead of 2D ultrasound, but the quality of this evidence was also low.

Inadvertent arterial puncture was reduced by 72% through use of 2D ultrasound (22 trials with 4,388 participants; RR 0.28; 95% CI: 0.18 to 0.44; P-value < 0.00001; I² = 35%). Use of 2D ultrasound reduced significant hematoma formation by 73% (13 trials with 3,233 participants; RR 0.27; 95% CI: 0.13 to 0.55; P-value 0.0004; I² = 54%). However, the quality of the evidence was very low. For Doppler ultrasound, this outcome was described in only one trial, thus making statistical analysis impossible.

The results relating to other complications such as thrombosis, embolism, hemomediastinum and hydromediastinum, hemothorax and hydrothorax, pneumothorax, subcutaneous emphysema and nerve injury were better (decrease of 66%) through use of 2D ultrasound (11 trials with 3,042 participants; RR 0.34; 95% CI: 0.15 to 0.76; P-value 0.009; I² = 17%), with moderate quality of evidence. In Doppler ultrasound trials, these outcomes were not reported.

#### Conclusions of this study

This systematic review^13^ suggested that use of 2D ultrasound in relation to venous catheter insertion into the internal jugular vein improves the results and diminishes adverse events, with very low to moderate quality of evidence. Use of Doppler ultrasound was better at the first attempt, with no difference in other outcomes. These results should be used with caution because of the quality of the present evidence and heterogeneity.

### 3. Ultrasound guidance versus anatomical landmarks for subclavian or femoral vein catheterization

Similarly to the review by Brass et al.,^13^ this systematic review^14^ addressed central venous catheter implantation and its complications, according to the techniques of the procedure. The primary objective was to evaluate the safety and effectiveness of 2D imaging ultrasound or Doppler ultrasound to guide puncture for insertion of central venous catheters, this time via the subclavian vein, axillary vein and femoral vein. The secondary objectives were the same as those of the previous study: to ascertain differences in effects between 2D ultrasound and Doppler ultrasound; differences between real-time and indirect puncture techniques; and possible distinctions between different groups of patients or different levels of experience among the persons responsible for insertion of the catheter. Thirteen studies were selected, enrolling 2,341 participants and 2,360 procedures. Unclear risk of bias was mentioned for almost all of the studies and heterogeneity was substantial, according to the review authors.

#### Main findings

For subclavian/axillary vein cannulation, the quality of the evidence was low regarding the overall success rate. There was no evidence that use of 2D ultrasound or Doppler ultrasound-guided puncture techniques made any difference in this outcome (RR 1.05; 95% CI: 0.97 to 1.13; P-value 0.22; I² = 78%). However, for femoral vein catheterization, a small increase in the overall success rate was reported (RR 1.11; 95% CI: 1.00 to 1.23; P-value 0.06; I² = 50%) with moderate quality of evidence.

For subclavian/axillary vein cannulation, there was no evidence of any difference between landmark and 2D real-time puncture ultrasound regarding the number of attempts needed to succeed (MD -0.38; 95% CI: -1.26 to 0.50; P-value 0.39; I² = 92%). However, the quality of the evidence was very low. For femoral vein catheterization, this outcome was reported in only one trial.

There was no evidence of any difference in the time taken to achieve successful cannulation for the subclavian/axillary vein (MD 10.48 seconds; 95% CI: -56.92 to 77.87; P-value 0.76; I² = 81%), but the quality of the evidence was low. For femoral vein catheterization, only one trial could be analyzed.

There was no evidence of any difference in success at the first attempt (RR 1.08; 95% CI: 0.85 to 1.36; P-value 0.53; I² = 0%) regarding subclavian/axillary vein cannulation, with high quality of evidence. However, ultrasound used during puncture of the femoral vein increased the rate of success at the first attempt (RR 1.73; 95% CI: 1.34 to 2.22; P-value < 0.0001; I² = 31), and the quality of the evidence was high.

#### Complications

For subclavian/axillary vein cannulation, use of 2D ultrasound or Doppler ultrasound-guided puncture techniques did not show any difference in the total perioperative and postoperative complication rate (RR 0.52; 95% CI: 0.23 to 1.17; P-value 0.11; I² = 60%), although the quality of the evidence was very low. For femoral vein catheterization, this outcome was reported in only one trial.

For subclavian/axillary vein cannulation, real-time ultrasound significantly reduced the risk of arterial puncture (RR 0.21; 95% CI: 0.06 to 0.82; P-value 0.02; I² = 0%), but the quality of the evidence was low. No evidence of any difference was found in relation to femoral vein catheterization (RR 0.40; 95% CI: 0.14 to 1.16; P-value 0.09; I² = 39%). However, the quality of the evidence was low.

Real-time ultrasound significantly reduced the risk of hematoma in subclavian/axillary vein cannulation (RR 0.26; 95% CI: 0.09 to 0.76; P-value 0.01; I² = 0%), with moderate quality of evidence. None of the trial authors reported this outcome for femoral vein catheterization.

Regarding use of 2D ultrasound or Doppler ultrasound-guided puncture techniques for subclavian/axillary vein cannulation, the study did not find evidence of any difference in this outcome (RR 0.29; 95% CI: 0.07 to 1.21; P-value 0.09; I² = 60%). However, the quality of the evidence was very low. Likewise, for femoral vein catheterization, no evidence of any difference was found (RR 0.49; 95% CI: 0.11 to 2.12; P-value 0.34; I² = 0%), and the quality of the evidence was low.

#### Conclusions of this study

2D ultrasound improves the safety and quality, compared with an anatomical landmark technique for the subclavian or femoral vein, but the results are uncertain.

### 4. Ultrasound-guided arterial cannulation for pediatrics

Ultrasound guidance may be useful not only for central venous access, but also in arterial and peripheral cannulation.^15^ Thus, this systematic review^15^ differed from the others included in this overview, since it addressed arterial cannulation (not venous) and involved only pediatric patients. Before catheterization, the artery could be located using palpation or Doppler auditory assistance; this was considered to be the control group. Participants subjected to 2D ultrasound-guided puncture for arterial puncture formed the intervention group. The study aimed to evaluate success rates and complication rates between these methods at potential sites for arterial cannulation (right or left radial, ulnar, brachial, femoral or dorsalis pedis artery). Five studies were included, reporting 444 arterial cannulations in accordance with the selection criteria. The results were presented as comparisons of 2D ultrasound guidance versus palpation or Doppler auditory assistance.

#### Main findings

Ultrasound guidance was found to significantly increase the success rate of cannulation at the first attempt (RR 1.96; 95% CI: 1.34 to 2.85) and increased successful radial artery cannulation within the first two attempts (RR 1.78; 95% CI: 1.25 to 2.51; P = 0.002). The quality of the evidence was moderate for both outcomes. However, ultrasound guidance did not significantly improve the rate of successful cannulation in comparison with palpation (RR 1.15; 95% CI: 0.95 to 1.40; P = 0.16).

The review authors were unable to perform a meta-analysis on the time taken for successful cannulation and the number of cannulas used. The number of attempts required for successful cannulation was presented in two studies, but no meta-analysis was possible.

The review authors did not conduct a sensitivity analysis. However, results regarding the need for assistance from another operator (i.e. the primary operator failed when attempting to insert the cannula and asked for help) were presented in one of the studies. A rate of 30.6% was reported in the ultrasound group, versus 33.7% in the palpation group (P = 0.73; 152 catheters).

#### Complications

The rate of complications such as hematoma was significantly reduced when using 2D ultrasound guidance during radial artery cannulation (RR 0.20; 95% CI: 0.07 to 0.60). The quality of the evidence was moderate. No studies reported any data on ischemic damage.

#### Conclusions of this study

There is evidence of moderate quality to support the use of ultrasound guidance for radial artery cannulation. Improved success rates at the first and second attempts were identified, along with lower complication rates, compared with the other techniques. Improved success rates at the first try may be more pronounced among infants and young children.

## DISCUSSION

The overall analysis on the reviews included suggested that use of 2D ultrasound-guided vascular access provided benefits, compared with use of anatomical landmarks and the palpation technique alone.

Reduction in the total rate of perioperative and postoperative complications/adverse effects was found in one of the reviews in relation to internal jugular vein catheterization using 2D ultrasound. Hematoma formation was reduced through application of ultrasound in all of the four SRs included. However, none of these reviews addressed the impact of use of ultrasound on patient discomfort and mortality. Nor did they evaluate the impact of different types of devices (e.g. point-of-care versus standard devices) and the skill with which these instruments are applied.

The major limitation of this overview was the small number of reviews included. Another limitation was that one of the reviews was out of date in the sense that it did not evaluate the evidence using the GRADE approach.^12^ This imposed limits on comparison of the evidence with that of the other reviews. Another issue was the low certainty of the evidence regarding most of the outcomes and the lack of evidence regarding arterial puncture in adults. Over recent decades, use of endovascular procedures has increased, and use of arterial accesses in adults, frequently via the radial or femoral artery, has become a routine procedure. Use of other arterial access points, such as the radial artery, especially in intensive care units for hemodynamic evaluation or blood sample collection, has given rise to concerns regarding avoidance of adverse events, considering that puncture may be performed frequently.

Nevertheless, all of the reviews included suggested that use of ultrasound for guided vascular access provided various benefits, compared with use of anatomical landmarks (vein puncture) and palpation (artery puncture) alone.

Some of the most recent clinical practice guidelines^16^^,^^17^^,^^18^ assessed the outcomes from ultrasound-guided vascular access only superficially or did not assess these outcomes, and they did not include any of the Cochrane SRs evaluated here.^12^^,^^13^^,^^14^^,^^15^^,^^16^^,^^17^^,^^18^ Therefore, the results from the present review may also serve to improve the next versions of the guidelines relating to vascular access.^16^^,^^17^^,^^18^

## CONCLUSION

Even with limitations regarding the quality of evidence, all of the four Cochrane reviews included in this overview showed that ultrasound guidance for vascular access provided some benefits. There is a lack of information regarding ultrasound-guided arterial puncture in adults. Therefore, further well-designed and well-conducted studies from which solid conclusions can be reached are still imperative, especially regarding arterial ultrasound-guided access. Additional evidence with high certainty regarding ultrasound guidance for venous and arterial puncture is needed in order to build up a robust body of evidence in this setting.
